# Late events after anti-CD19 CAR T-cell therapy for relapsed/refractory B-cell non-Hodgkin lymphoma

**DOI:** 10.3389/fonc.2024.1404351

**Published:** 2024-06-11

**Authors:** Ana Costa Cordeiro, George Durisek, Marjorie Vieira Batista, Jayr Schmidt, Marcos de Lima, Evandro Bezerra

**Affiliations:** ^1^ Hematology Division, AC Camargo Cancer Center, São Paulo, SP, Brazil; ^2^ College of Medicine, The Ohio State University, Columbus, OH, United States; ^3^ Infectious Disease Division, AC Camargo Cancer Center, São Paulo, SP, Brazil; ^4^ Division of Hematology, The Ohio State University, Columbus, OH, United States

**Keywords:** chimeric antigen receptor T-cells (CART), lymphoma, long-term follow up, late complications, cytopenia, hypogammaglobulinemia, infection, secondary malignancies

## Abstract

**Background:**

The short-term complications from chimeric antigen receptor T-cell therapy (CART) are well characterized, but the long-term complications still need to be further investigated. Therefore, herein, we will review the currently available literature published on the late adverse events following CART.

**Methods:**

We reviewed published data available from pivotal trials and real-world experiences with anti-CD19 CART (CART19) for adults with lymphoma. We defined late events as occurring or persisting beyond 1 month after CART infusion. We focused our literature review on the following late-event outcomes post-CART19: cytopenia, immune reconstitution, infections, and subsequent malignancies.

**Results:**

Grade 3–4 cytopenia beyond 30 days occurs in 30%–40% of patients and beyond 90 days in 3%–22% of patients and is usually managed with growth-factor and transfusion support, along with neutropenic prophylaxis. B-cell aplasia and hypogammaglobulinemia are expected on-target off-tumor effects of CART19, 44%–53% of patients have IgG < 400 mg/dL, and approximately 27%–38% of patients receive intravenous immunoglobulin (IVIG) replacement. Infections beyond the initial month from CART19 are not frequent and rarely severe, but they are more prevalent and severe when patients receive subsequent therapies post-CART19 for their underlying disease. Late neurotoxicity and neurocognitive impairment are uncommon, and other causes should be considered. T-cell lymphoma (TCL) after CART is an extremely rare event and not necessarily related to CAR transgene. Myeloid neoplasm is not rare post-CART, but unclear causality given heavily pretreated patient population is already at risk for therapy-related myeloid neoplasm.

**Conclusion:**

CART19 is associated with clinically significant long-term effects such as prolonged cytopenia, hypogammaglobulinemia, and infections that warrant clinical surveillance, but they are mostly manageable with a low risk of non-relapse mortality. The risk of subsequent malignancies post-CART19 seems low, and the relationship with CART19 and/or prior therapies is unclear; but regardless of the possible causality, this should not impact the current benefit–risk ratio of CART19 for relapsed/refractory B-cell non-Hodgkin lymphoma (NHL).

## Introduction

Chimeric antigen receptor T-cell therapy (CART) has been approved by the Food and Drug Administration (FDA) since 2017 as the standard of care (SOC) for the treatment of many relapsed/refractory B-cell or plasma cell malignancies and provides durable remission and long-term survival for a subset of patients (1–13). Because it is the first gene therapy approach-based therapy approved by the FDA as SOC, there is a need for long-term follow-up of adverse events following CART to better delineate the possible late complications of this groundbreaking therapy.

The short-term complications of CART such as cytokine release syndrome (CRS), immune effector cell-associated neurotoxicity syndrome (ICANS), infection risk, myelotoxicity, and hemophagocytic lymphohistiocytosis (HLH) are well characterized (14–16). However, prolonged cytopenia, B-cell aplasia (BCA), and late infections, which are established long-term complications of CART, still need to be further characterized in larger cohorts with longer follow-ups (17). On top of a better understanding of these known long-term complications of CART, there is also interest in investigating the risk of secondary malignancies following CART. Therefore, herein, we will review the currently available literature published on the long-term follow-up and late events following CART.

## Materials and methods

### Study population

We reviewed published data available through PubMed search from pivotal trials and real-world experiences with anti-CD19 CART (CART19) for adults with lymphoma. We focused our review on CART19 for lymphoma, as these are the longest available products, since 2017, and have the longest follow-up and a lower rate of relapse and/or post-CART as allogenic transplant consolidations compared to other diseases and/or products.

### Endpoints

We defined late events as occurring or persisting beyond 1 month after CART infusion. We focused our literature review on the following late-event outcomes post-CART19: cytopenia, immune reconstitution, infections, and subsequent malignancies.

## Results/discussion

### Late cytopenia

The terminology for cytopenia after CAR T-cell therapy varies among studies. Usually, it is called early cytopenia for those occurring up to 30 days, “short-term” for those occurring between 30 and 90 days, and persistent or late for those occurring after 90 days (17–19). The term prolonged cytopenia was used for cytopenia beyond 21, 28, 30, and 90 days. Delayed cytopenia is used for those occurring after an initial recovery (19). Overall, grade 3–4 cytopenia beyond 30 days occurs in 30%–40% of patients and beyond 90 days in 3%–22% of patients, with neutropenia and thrombocytopenia more common than anemia (18).

In the pivotal clinical trials of third-line (3L) CART19 for relapsed/refractory (R/R) large B-cell lymphoma (LBCL), the prevalence of grade 3/4 (G3/4) cytopenia beyond day 90 (D90) ranged from 17% to 38% (2–4, 20–22). When looking at the real-world evidence (RWE) data of 3L CART19 for R/R LBCL through the Center for International Blood and Marrow Transplant Research (CIBMTR) registry studies, the prevalence of grade 4 neutropenia and/or thrombocytopenia at day 30 (D30) ranged from 10% to 22%. Among the second-line (2L) CART19 trials for R/R LBCL, the prevalence of G3/4 cytopenia at D35 was 43% with liso-cel and 29% at D30 with axi-cel, but at 6 months, the prevalence was only 5% with axi-cel and not available for liso-cel (**Table 1**) (5, 6, 26).

**Table 1:** Late cytopenia after CART19.

Reference	Product	N, disease	Late cytopenia (≥90 days)
Pivotal studies			
ZUMA-1 (3, 21)	3L Axi-cel	108, LBCL	D90 G3/4 cytopenia, 17% (11% neutropenia, 7% thrombocytopenia, and 3% anemia)
JULIET (4, 20)	3L Tisa-cel	111, LBCL	D90 G3/4 cytopenia, 38% (38% thrombocytopenia and 0% neutropenia)
TRANSCEND (2, 22)	3L Liso-cel	269, LBCL	D90 G3/4 thrombocytopenia, 22%; neutropenia, 7%; and anemia, 2%
ZUMA-2 (11, 23)	3L Brexu-cel	68, MCL	G3/4 cytopenia at D90, 26% (neutropenia 16%, thrombocytopenia 16%, and anemia 12%)
ZUMA-5 (7, 24)	3L Axi-cel	124, FL	D30 G3/4 cytopenia, 33%; D90, 6%; 1 year, 4%; 2 years, 3%
ELARA (1, 25)	3L Tisa-cel	97, FL	Probability of G3/4 cytopenia at 6 months, 18%; 2 years, 3.3% (prevalence not provided)
ZUMA-7 (6, 26)	2L Axi-cel	170, LBCL	G3/4 cytopenia D30, 29%; 6 months, 5%; 1 year, 2%
TRANSFORM (5)	2L Liso-cel	89, LBCL	G3/4 cytopenia D35, 43%
Real-world evidence studies			
Pasquini et al. (27)	3L Tisa-Cel	155, LBCL	G4 thrombocytopenia D30, 14%; D100, 11% G4 neutropenia D30, 7%; D100, 3%
Jacobson et al. (28)	3L Axi-cel	1297, LBCL	D30 G4 thrombocytopenia, 22% D30 G4 neutropenia, 7%
Crombie et al. (29)	3L Liso-cel	323, LBCL	D30 G4 neutropenia and/or thrombocytopenia cytopenia, 10%
Wang et al. (30)	3L Brexu-cel	168, MCL	G3/4 neutropenia D30, 33%; D90, 18% G3/4 thrombocytopenia D30, 43%; D90, 11%
Bachy et al. (31)	3L Axi-cel and tisa-cel	729, LBCL	D90 G3/4 cytopenia: axi-cel, 12%, vs. tisa-cel, 4%

CART19, CD19 chimeric antigen receptor T-cell therapy; 3L, third line; 2L, second line; LBCL, large B-cell lymphoma; MCL, mantle cell lymphoma; FL, follicular lymphoma.

The prevalence of G3/4 cytopenia at D90 was 26% in the pivotal trial of brexucabtagene autoleucel (brexu-cel) for R/R mantle cell lymphoma (MCL), and in the RWE, the prevalence of G3/4 neutropenia and thrombocytopenia at D90 was 18% and 11%, respectively. In pivotal trials of CART19 for R/R follicular lymphoma (FL), the prevalence of D90 G3/4 cytopenia was 6% with axi-cel, and the probability of G3/4 cytopenia at 6 months was 18% with tisa-cel (**Table 1**) (1, 7, 11, 23–25, 30).

Because of the significant variability of how post-CART cytopenia is reported within these different studies, it is not possible to draw a conclusion if cytopenia is more prevalent in the real-world vs. pivotal trials, 2L vs. 3L products, or between different products. However, some studies show a trend for a higher incidence of late cytopenia with axi-cel compared to tisa-cel. In the French registry DESCAR-T, grade 3–4 cytopenia beyond 90 days was seen in 11.5% and 3.8% of patients treated with axi-cel versus tisa-cel, respectively, with neutropenia 8.6 vs. 2.9% and thrombocytopenia 8.6% vs. 1.9% (31). Of note, lymphodepletion intensity was higher for axi-cel compared to tisa-cel (**Table 1**) (3, 4).

Reported risk factors for prolonged cytopenia are baseline cytopenia, prior treatments including bone marrow transplant, and factors related to inflammation such as C-reactive protein (CRP), ferritin, and other inflammatory markers (19, 32). Although not specific for cytopenia lasting more than 90 days, the CAR-HEMATOTOX score is interesting in predicting the risk of longer duration of neutropenia and higher incidence of severe thrombocytopenia and anemia, using baseline parameters of platelet count, hemoglobin, absolute neutrophil count (ANC), CRP, and ferritin. It was based on a large multicenter study of 235 patients treated with axi-cel and tisa-cel in a real-world setting that showed that the risk factors for late cytopenia were hematopoietic reserve and baseline inflammation (19, 32).

The impact of prolonged cytopenia is great because of infection risk (33, 34), transfusion support (33), healthcare resources for laboratory monitoring through hospitalization (18), and therapeutic limitations to treat disease relapse, especially in the clinical trial setting (35). Also, some studies tried to correlate late cytopenia with outcomes such as response, progression-free survival (PFS), and overall survival (OS), but this is not well-established (36, 37).

The etiology of cytopenia occurring beyond 30 and 90 days after CART is not completely understood but is possibly related to immune or inflammatory-mediated alterations in the bone marrow microenvironment and suppression of hematopoietic stem cell (HSC) (18). CRS plays a role that is more pronounced for early and short-term cytopenia (38, 39). When obtained, the bone marrow is usually hypocellular, and Strati et al. identified clonally expanded CX3CR1^hi^ cytotoxic T cells expressing IFN-gamma in the bone marrow of patients with prolonged cytopenia beyond 30 days, suggesting a possible IFN-γ-driven hematopoiesis impairment (40).

Based on the role of IFN-γ-driven hematopoiesis impairment post-CART, the pre-clinical effect of thrombopoietin receptor agonist (TPO-RA) preserving hematopoiesis in IFN-γ-mediated inflammatory conditions (41), and their effectiveness in aplastic anemia, TPO-RA have been used for the treatment of post-CART cytopenia (42–44). However, it is challenging to measure TPO-RA effectiveness in post-CART cytopenia without a randomized clinical trial, as in most cases, cytopenia will eventually improve without specific interventions.

Lastly, there are reports of the use of autologous and allogeneic stem cell boost (SCB) for patients with lymphoma with very prolonged and profound cytopenia after CART (45, 46). Rejeski et al. reported 12 cases where the median time of SCB infusion was 69 days after CART19, and the median time to neutrophil and platelet engraftment was 15 and 21 days, respectively (45). Gagelmann et al. reported SCB at a median 43 days after CART19 with neutrophil recovery in 84% and a median time to recovery of 9 days from SCB (46). Allogeneic hematopoietic CD34+ selected stem cell boost was also reported in seven patients with B-cell acute lymphoblastic leukemia (B-ALL) (47), and allogeneic transplant was reported for a patient with lymphoma, prolonged cytopenia, and ASXL1 mutation (48).

#### Take-home messages

Most cases of cytopenia will resolve with time; therefore, intervention should be considered only for patients with persistent and clinically relevant cytopenia.

#### Recommendations

More frequent blood count monitoring is recommended for patients with high CAR-HEMATOTOX beyond 30 days from infusion, which is recommended to be gradually spaced out as appropriate based on the counts, for example, weekly between D30 and D60, then bi-weekly between D60 and D90, and monthly between D90 and D180. When evaluating a patient with cytopenia beyond 30–90 days after CART, the first step is to rule out drug effects and infections. HLH is rarely seen beyond 30 days from infusion and outside acute complications of CART. Bone marrow evaluation is indicated for patients with prolonged and significant cytopenia to rule out therapy-related myeloid neoplasm and underlying disease relapse with bone marrow involvement (18).

There is no evidence-based standard of care in how to manage prolonged cytopenia post-CART, and the approach should be tailored to the individual patient. Clinical trials should be prioritized if available, but in the absence of a clinical trial, consider growth-factor support, either granulocyte colony-stimulating factor (G-CSF) and/or TPO agonists, when patients have persistent severe clinically significant cytopenia. Also, consider bacterial/fungal infection prophylaxis in persistent severe neutropenia, especially in those with a history of recurrent/opportunistic infection. SCB, when available, can be considered for patients with persistent severe clinically significant cytopenia despite growth-factor support, but ideally in an investigational setting (18).

### Immune reconstitution

In addition to prolonged cytopenia, patients may have prolonged lymphopenia and hypogammaglobulinemia after CART19. B-cell aplasia is an expected on-target off-tumor effect and correlates with CART persistence (20, 49). Sustained polyclonal B-cell recovery was reported after tisa-cel in patients with long-duration complete responses (20) and was reported in 50% of patients with complete responses at a median time of 6.7 months after tisa-cel infusion (20). After treatment with axi-cel, approximately 30%, 40%, 50%, and 75% of patients had detectable B cells after 6 months, 12 months, 18 months, and 24 months of treatment, respectively (3, 50). Baird et al. suggested that the duration of B-cell aplasia correlates with response and that loss of B-cell aplasia is concurrent with or precedes clinical or radiological relapse after axi-cel (50).

Hypogammaglobulinemia is an expected effect after the depletion of B cells. Although 20%–30% of patients had low IgG levels even before CART (17, 51), IgG levels are expected to decline by 17% mean decrement in the first 100 days (49) and reach a nadir approximately 6 months after CART (17, 34, 49, 51). Up to 60% of patients had low IgG levels at 3 months after infusion, and up to 50% still had IgG < 400 mg/dL at 1 year without intravenous immunoglobulin (IVIG) replacement (51). The use of IVIG in clinical practice is highly variable among different institutional guidelines, so the percentage of patients requiring IVIG replacement varies from 27% to 38% (**Table 2**). Of note, among the pivotal CART19 trials in non-Hodgkin lymphoma (NHL), the cumulative rate of use of IVIG seems lower when CART19 is used as 2L therapy, 13%–17%, vs. 3L therapy, 30%–38%, probably due to cumulative effect from prior therapies (**Table 2**) (1, 3–7, 23).

**Table 2:** Hypogammaglobulinemia after CART19.

Reference	Product	N	Hypogammaglobulinemia
Pivotal studies			
ZUMA-1 (3)	**3L Axi-cel**	**108 LBCL**	31% required IVIG, while 44% among with ongoing response
JULIET (4)	**3L Tisa-cel**	**111 DLBCL**	30% required IVIG
TRANSCEND (22)	**3L Liso-cel**	**269 DLBCL**	Hypogammaglobulinemia any grade 14%
Zuma-2 (23)	**3L Brexu-cel**	**68 MCL**	38% of patients received IVIG
Zuma-7 (6)	**2L Axi-cel**	**170, LBCL**	IVIG in 17% of patients who received axi-cel
TRANSFORM (5)	**2L Liso-cel**	**89, LBCL**	IVIG in 13% of patients who received liso-cel
Zuma-5 (7)	**3L Axi-cel**	**124, FL**	33% required IVIG
ELARA (1)	**3L Tisa-cel**	**97, FL**	34% required IVIG
Real-world evidence studies			
Wudhikarn et al. (52)	**Axi-cel and tisa-cel**	**60 NHL**	Pre-conditioning IgG < 400 in 25%. Hypogammaglobulinemia in 43.8% at 30 days and 81% at any time. 31.7% received IVIG.
Barmettler et al. (53)	**Axi-cel and tisa-cel**	**101 NHL**	IgG < 400 mg/dL in 23% pre-conditioning and 69% after CART. Median time to IgG nadir 2 months.
Baird et al. (50)	**Axi-cel**	**41 NHL**	Pre-conditioning 23.5% had IgG < 400 mg/dL. Over the first 9 months, 62.2% had IgG < 400 mg/dL. Among patients with normal baseline IgG, 30% developed hypogammaglobulinemia after a median of 2 months. At 18 months, 44% still had hypogammaglobulinemia. 37% received IVIG after a median of 160 days.
Logue et al. (51)	**Axi-cel**	**85 NHL**	Pre-conditioning IgG < 400 mg/dL in 43%. IgG levels reached a nadir 6 months after infusion. 53% had IgG < 400 mg/dL at 1 year. New-onset hypogammaglobulinemia after CART in 59% at 1 month and 74% cumulatively. 27% received IVIG.

CART19, CD19 chimeric antigen receptor T-cell therapy; 3L, third line; 2L, second line; IVIG, intravenous immunoglobulin; LBCL, large B-cell lymphoma; DLBCL, diffuse large B-cell lymphoma; MCL, mantle cell lymphoma; FL, follicular lymphoma; NHL, non-Hodgkin lymphoma.

Beyond B-cell aplasia, CD4+ T-cell count normalization can take more than 1 year, with a median CD4 count of 150–250 cells/mm^3^ at 1 year post-infusion (51, 52) but can remain as low as 150 cells/mm^3^ at 18 months. Risk factors for delayed CD4 reconstitution are older age, which may be related to thymic function and recovery in the CD19 compartment due to the participation of B cells in T CD4+ expansion (50). The practical impact of following CD4 counts is the possible increased risk of infections and reduced immune response to vaccines. It is important to note that CD4 is <200 cells/mm^3^ in up to 40% of patients before lymphodepletion (52). In contrast, CD56+ NK cells and CD8+ T lymphocytes recover faster and are normalized in most patients at 1 year (33, 50, 51).

#### Take-home messages

B-cell aplasia, low CD4 counts, and hypogammaglobulinemia are expected events after CD19 CAR T-cell infusion, with a possible increased risk of infections and reduced immune response to vaccines. Of note, many patients already have these immunologic impairments prior to conditioning for CART19 due to prior therapies for NHL. At 1 year post-infusion, B-cell recovery occurs in approximately 50% of patients, the median CD4 count is 150–250 cells/mm^3^, and 44%–53% of patients have IgG < 400 mg/dL. Approximately 27%–38% of patients receive IVIG replacement.

#### Recommendations

Baseline evaluation of IgG levels prior to CART19 and monthly during the initial 3 months after CART19 is recommended and spaced out accordingly until B-cell recovery. Hill et al. recommended IVIG replacement after CART for patients with IgG <400 mg/dL or <600 mg/dL with serious, persistent, or recurrent infections within 3 months from CART (54) and beyond the initial 3 months only if IgG < 400 mg/dL with serious, persistent, or recurrent infections, or in case of ongoing B-cell aplasia, <20 B cells/mm^3^ (54).

Prior to stopping IVIG replacement, it is recommended to confirm B-cell recovery because IgG levels may be artificially normalized due to IVIG. CD4 count monitoring is also recommended post-CART19 prior to stopping *Pneumocystis jiroveciijiroveci* pneumonia (PJP), *Herpes simplex virus* (HSV), and Varicella zoster virus (VZV) prophylaxis. Lastly, it is recommended to re-check IgG and CD4 counts after the start of post-CART19 systemic therapy for NHL, as levels may decline again despite initial immune reconstitution post-CART19 (54).

### Late infections

Late infections are defined as infections beyond day 30 after CART. Initial long-term follow-up data of 54 patients regarding infection beyond 90 days after CART19 reported an infection density of 0.55 infection/100 days at risk (2.08 per patient-year) (17). The most common infections were upper respiratory tract infections (48%), followed by lower respiratory infections (23%), 80% of patients were treated on an outpatient basis, 20% were inpatients, and 5% were in the intensive care unit (ICU) (17). Wudhikarn et al. showed that in 60 NHL patients treated with tisa-cel or axi-cel, most infections occurred within the first 2 months after CART (52). Decreasing incidence and the grade of infection over time are consistently reported (**Table 3**) (34, 50, 51).

**Table 3:** Late infection after CART19.

Reference	Product	N	Late infection
Cordeiro et al. (17)	CART19 JCAR014	54 (NHL, B-ALL, and CLL)	Infection density beyond D90 from CART19 was 0.55 infection/100 patient-days at risk (2.1 per patient-year). Upper respiratory tract infections (48%), lower respiratory infections (23%), 80% managed as outpatients, 20% as inpatients, and 5% in ICU
Wudhikarn et al. (52)	Axi-cel and tisa-cel	60 NHL	Absolute number of infections post-CART19: 101 events. 23% between D30 and D100, 14% between D100 and D180, and 27.5% after D180
Baird et al. (50)	Axi-cel	41 NHL	Infection density through D28, 2.35; between D29 and D180, 0.38; D181–D365, 0.46; and beyond 1 year, 0.33 (#/100 patient-days)
Logue et al. (51)	Axi-cel	85 NHL	Infection rate 11.7 per 1,000 person-days within D30 from CART19, while 2.3 per 1,000 person-days between D30 and D90

CART19, CD19 chimeric antigen receptor T-cell therapy; NHL, non-Hodgkin lymphoma; B-ALL, B-cell acute lymphoblastic leukemia; CLL, chronic lymphocytic leukemia; ICU, intensive care unit.

#### Risk factors for infections

The risk factors for early and late infections are different. Baseline immune impairment after many lines of therapy including hematopoietic cell transplant (HCT), neutropenia, CRS (34, 55), and use of steroids (50, 52, 56) are important risk factors in the early phase. In comparison, impaired immune reconstitution, including persistent CD19+ B-cell aplasia and hypogammaglobulinemia (57), and prolonged cytopenia are more important in the late phase (50). Approximately 15%–70% of patients treated with tisa-cel and axi-cel received tocilizumab for treatment of CRS; however, the use of a short course of tocilizumab is not associated with an increased risk of infections (58). One important risk factor is disease relapse/progression and subsequent therapies post-CART19; for example, 35% of patients undergoing allogeneic HCT post-CART19 died from infection compared to 30% from disease-related deaths post-transplant (59).

#### Bacterial infection

Bacterial infection is more common in the first month and up to 3 months after CART19. The incidence of severe bacterial infection beyond 90 days is low (52). However, based on studies evaluating the retained antibodies against vaccine-preventable infections, such as *Streptococcus pneumoniae* and *Haemophilus influenzae*, IgG titers seem to be lost after CART19. This suggests that those patients could be at risk for encapsulated bacterial infection, mainly in the settings of prolonged hypogammaglobulinemia and B-cell aplasia (49, 60, 61).

#### Viral infections

Most late infections are mild viral respiratory tract infections (17, 52). COVID-19 was a particular concern and was reported in approximately 5% of patients (in a population not vaccinated for COVID-19 and including relapsed disease), with a median time between infusion and COVID-19 infection of approximately 6 months (62); infection was asymptomatic, mild, and severe in 10%, 20%, and 67%, respectively, of patients, and the expected mortality in this population is approximately 50% (62, 63).

Cytomegalovirus (CMV) reactivation occurs in 1%–2% of patients and is usually in the early phase since the major risk factor for CMV reactivation is steroid use (64), but most cases are CMV viremia without CMV disease (52, 64, 65). In contrast, there is currently no formal indication for CMV monitoring, guidelines, and local practices for infection prevention in CAR T-cell therapy recipients from France, the USA, and Switzerland that recommend CMV monitoring in seropositive patients at high risk, such as those receiving steroids for more than 3 days or more than five doses of dexamethasone. Monitoring is typically conducted weekly until 1 month after the last dose of steroids. Although the preemptive CMV standard threshold is yet to be determined, as a local recommendation, Fred Hutch (USA) suggests a threshold of 150 IU/mL in plasma (54, 66–69).

Human herpesvirus 6 (HHV-6) was also reported to be predominant in the first month after CART19 (70, 71). In contrast, HSV and VZV reactivation is reported even beyond 1 year (50). Progressive multifocal leukoencephalopathy caused by JC virus was reported in three patients after CART19, diagnosed 7, 14, and 24 months after CART, and associated with severe hypogammaglobulinemia and low CD4 (72–74).

#### Fungal infections

Fungal infections are reported in 1%–13% of patients. Early fungal infections are usually caused by *Candida* spp and *Aspergillus* spp, while PJP and coccidioidomycosis are reported later (75). Risk factors for late fungal infections include steroid use, low CD4 counts, discontinuation of PJP prophylaxis (52), and previous and current environment exposure, especially for mold and endemic fungi.

#### Vaccination

So far, there is no definite evidence that revaccination is beneficial after CART19 cell treatment. IgG total levels are not a good surrogate for pathogen-specific IgG levels. Studies show that individuals can keep protective levels against pathogens such as HSV (51), and measles (49), and even if immunogenic antigen-specific IgG levels decreased, they rarely fell below 50% of their pretreatment baseline (50). Seroprotection preservation occurs despite B-cell aplasia and hypogammaglobulinemia possibly due to the fact that the long-living plasma cells are CD19 negative, so they are spared from CART19 (61). Pathogen-specific IgG levels were similar for most pathogens in patients post-CART19 with or without immunoglobulin replacement, except for mumps, hepatitis A virus (HAV), hepatitis B virus (HBV), *H. influenzae*, *S. pneumoniae*, and *Bordetella pertussis* (60). Interestingly, IgG titers for vaccine-preventable infection were similar to those in the healthy population. Hill et al. also showed that influenza vaccination antibody responses can occur post-CART19 and are not impacted by severe hypogammaglobulinemia or B-cell aplasia (49). In contrast, the optimal timing of vaccination after CAR T-cell therapy to achieve peak immunogenicity remains to be determined, and its role in preventing the acquisition and severity of infections is well-established. As of the present, there are no specific immunization guideline recommendations. However, some studies have indicated positive responses to the inactivated influenza vaccine both before and after CAR T-cell therapy, even in cases of hypogammaglobulinemia or B-cell aplasia. For instance, Walti et al. demonstrated evidence of immunogenicity within 30 days before and 30–90 days after CAR T-cell therapy, suggesting that this could serve as evidence to consider initiating influenza immunization (76).

#### Take-home messages

Infections are more prevalent in the first month after CART19, the incidence declines over time, and it is a rare severe infection beyond the initial month from CART19. Early infections are more severe and mostly bacterial, in contrast with infections after 3 months, which are milder and mostly viral respiratory tract infections. Of note, patients who need further systemic therapies for NHL post-CART19 may be at higher risk of infection than usual for that type of therapy (59).

### Recommendations

We recommend prophylaxis for HSV/VZV and PJP for at least 12 and 6 months from CART19, respectively, and to check CD4 counts prior to discontinuation. We recommend neutropenic prophylaxis with levofloxacin and fluconazole while neutrophils are less than 0.5 × 10^9^/L. If prolonged severe neutropenia is more than 3 weeks, a mold-active azole should be considered. In patients with prior or chronic hepatitis B virus, entecavir should be considered for at least 6 months post-CART19 along with viral load monitoring (54).

### Subsequent malignancies

Given the genetic editing nature of CART19, since its development, there has been a theoretical concern about the potential risk of T-cell and other malignancies due to insertional mutagenesis CAR transduction. Because of this, the FDA required that all patients need to be followed up for 15 years after treatment to assess the long-term safety and the risk of secondary malignancies. Recently, the FDA reported the occurrence of T-cell malignancy after different CART products for different diseases, warranting further investigation despite that the overall benefits of CART continue to outweigh its potential risk (77–79).

To date, there have been few reports of T-cell lymphoma (TCL) after treatment with CAR T cells. There were two cases of CART-derived T-cell malignancy among 10 patients treated with an investigational CART product manufactured by piggyback, a non-viral technology (80). There is also a case report of documented B-cell maturation antigen (BCMA) CAR+ TCL following treatment with ciltacabtagene autoleucel (cilta-cel) where the CAR insertion in the 3′ untranslated region of *PBX2* along with other likely pre-existing genetic mutations (81). In another report from the University of Pennsylvania group where 449 patients received CART, only one case of TCL was reported after axi-cel, but quantitative PCR ruled out association with CAR transgene, and the T-cell clone preceded axi-cel (**Table 4**) (82).

**Table 4:** Subsequent malignancies after CART.

Reference	Description of subsequent malignancies
T-cell malignancy (80–82)	2 out of 10 patients treated with investigational CART19 product manufactured with piggyback developed CAR-driven T-cell lymphoma. 1 case of TCL after cilta-cel driven by CAR transgene insertion 1 case of TCL after axi-cel not associated with CAR transgene
Myeloid neoplasm (17, 33, 82–84)	Gurney et al., cumulative incidence at 3 years post-CART, 9% Ghilardi et al., cumulative incidence of any secondary hematologic malignancy at 5 years post-CART, 2.3% Cordeiro et al., 4.5% of the patients Strati et al., 13% of the patients
Solid tumors (84)	Ghilardi, cumulative incidence at 5 years, 15%; 5 out 12 cases of solid tumors were non-melanoma skin cancer Cordeiro et al., 7% of the patients had non-melanoma skin cancer

CART19, CD19 chimeric antigen receptor T-cell therapy; TCL, T-cell lymphoma.

Moreover, much more often seen than TCL, there are many reports of myeloid neoplasms following CART (17, 27, 28, 33, 83, 84), including a recent boxed warning from the FDA of cilta-cel because 10% of the patients from their pivotal trial CARTITUDE-1 developed myeloid neoplasms (13). It is unclear if the development of subsequent myeloid malignancies is related to receipt of CART itself or to the fact that this is a heavily pretreated population already at risk of therapy-related myeloid neoplasm and perhaps now is experiencing longer survival from their underlying disease due to CART (77–79). The Mayo Clinic group reported a cumulative incidence of myeloid neoplasm of 9% at 3 years post-CART, and 70% of the patients with baseline samples had clonal hematopoiesis prior to CART (84) and median time to diagnosis of 9 months from CART (83). Similarly, the Fred Hutchinson group had also reported that among four (5%) cases of myeloid neoplasm post-CART, two of them had cytogenetic abnormality preceding CART19 (**Table 4**).

Lastly, there are also several reports of solid tumors post-CART (17, 27, 28, 82, 85), with cumulative incidence at 5 years of 15% (82), but nearly half are non-melanoma skin cancer (**Table 4**).

#### Take-home messages

TCL after CART is an extremely rare event and not necessarily related to CAR transgene. Myeloid neoplasm is not rare post-CART, but unclear causality given heavily pretreated patient population is already at risk for therapy-related myeloid neoplasm.

#### Recommendations

Consider the differential diagnosis of myeloid neoplasm in patients with prolonged cytopenia after CART (33, 84). The benefits of CART19 in R/R NHL outweigh the risks of secondary malignancies.

### Neuropsychiatric disorders and neurocognitive function

There is a report of delayed neurotoxicity 6 months after CART, with confusion, disorientation, and expressive aphasia, in which extensive workup for cerebral ischemia and infection was normal and unresponsive to dexamethasone but with response to cyclophosphamide (86). There is one case report of ascending paresthesia due to transverse myelitis at day 27, with a relapsing/remitting course over a 5-month period, treated with steroid pulse, IVIG, and plasma exchange (87). There are case reports of other neuropsychiatric events such as cerebrovascular events, Alzheimer’s dementia, peripheral neuropathy (17), and memory impairment (ZUMA-1) probably unrelated to CART.

Neurocognitive function was impaired at baseline in 33% of patients (88). After CART, 47.5% of patients reported at least one cognitive difficulty and/or clinically meaningful depression and/or anxiety, with depression before CART associated with a higher likelihood of self-reported post-CART cognitive difficulties, and younger age associated with worse long-term global mental health, anxiety, and depression (89). Neurocognitive assessments after CART show a possible small transient decline at day 90 (90, 91) with similar neurocognitive function at mid/long-term (6–24 months) compared to baseline (92, 93).

#### Take-home messages

Late neurotoxicity is unexpected, and other etiologies should be considered. Most studies showed a small decline or no relevant neurocognitive impairment after CART.

#### Recommendations

It is recommended to counsel patients regarding possible changes in cognition and reinforce neuropsychological evaluation before and after CART.

## Conclusion

CART19 is associated with clinically significant long-term effects such as prolonged cytopenia, hypogammaglobulinemia, and infections, which warrant clinical surveillance, but they are mostly manageable with a low risk of non-relapse mortality. There is an unmet need to investigate strategies to prevent and treat post-CART19 prolonged cytopenia. The risk of subsequent malignancies post-CART19 seems low, and the relationship with CART19 or prior therapies is unclear; but regardless of the possible causality, this should not impact the current benefit–risk ratio of CART19 for relapsed/refractory B-cell NHL.

## Author contributions

AC: Conceptualization, Investigation, Methodology, Data curation, Writing – original draft. GD: Writing – review & editing. MB: Data curation, Investigation, Writing – review & editing. JS: Writing – review & editing. Md: Writing – review & editing. EB: Conceptualization, Investigation, Methodology, Supervision, Writing – review & editing.
